# Association of papilledema severity with retinal vessel tortuosity in idiopathic intracranial hypertension

**DOI:** 10.1007/s00417-026-07254-9

**Published:** 2026-05-02

**Authors:** Yuhang Zou, Farzan Abdolahi, Anthony E. Felder, Connie Huang, Kimberly K. Gokoffski, Mahnaz Shahidi

**Affiliations:** 1 Department of Ophthalmology, University of Southern California Keck School of Medicine, Los Angeles, CA USA; 2 Alfred E. Mann Department of Biomedical Engineering, University of Southern California, Los Angeles, CA USA; 3 Richard and Loan Hill Department of Biomedical Engineering, University of Illinois Chicago, Chicago, IL USA

**Keywords:** Papilledema, Retina, Vessel tortuosity, Idiopathic intracranial hypertension

## Abstract

**Purpose:**

Idiopathic intracranial hypertension (IIH) induces papilledema and can result in progressive visual dysfunction and retinal vascular changes. The purpose of the current study was to determine cross-sectional and longitudinal associations between papilledema severity and retinal vessel tortuosity (RVT) in IIH.

**Methods:**

Images from 24 eyes of 15 patients diagnosed with papilledema were acquired at two visits. Papilledema severity was graded by Frisen score (FS). Images were analyzed to determine RVT in arteries and veins within the peripapillary region using the first-order derivative method. Inter-visit differences in FS (ΔFS) and RVT (ΔRVT) were calculated. Generalized estimating equation models determined associations of FS and ΔFS with RVT and ΔRVT, respectively.

**Results:**

Based on compiled arterial or venous RVT data from both visits, there was no significant association between RVT and FS (*p* = 0.89), whereas venous RVT (0.31 ± 0.02; *N* = 48) was significantly higher than arterial RVT (0.24 ± 0.01; *N* = 48) (*p* = 0.03). Compared to the ΔFS = 0 group, venous ΔRVT was higher in the ΔFS = 3 group (β = 0.05, *p* = 0.005), while arterial ΔRVT was higher in ΔFS *≥* 1 (β *≥* 0.04; p *≤* 0.04).

**Conclusion:**

The finding of an association between improvement in papilledema and reduction in RVT shows promise for vessel tortuosity as a biomarker for monitoring disease progression and response to therapeutic interventions, as well as advancing knowledge of retinal vascular changes due to papilledema.

## Abstract (fr)

**Key messages:**

***What is known*:**

- Idiopathic intracranial hypertension can result in papilledema and visual dysfunction.

- Papilledema is linked to changes in retinal vascular morphology.

***What is new*:**

- Improvement in papilledema severity is associated with a reduction in retinal vessel tortuosity.

- Retinal vessel tortuosity shows promise as a biomarker for diagnosis and therapeutic intervention for papilledema and intracranial hypertension.

## Introduction

Idiopathic intracranial hypertension (IIH) is a disorder characterized by elevated intracranial pressure (ICP) and has a high prevalence in young obese women. IIH is commonly accompanied by papilledema [1–3]. IIH-induced papilledema can result in progressive vision dysfunction and permanent vision loss [2]. Although the vascular pathogenesis of IIH remains unclear, studies support the critical role of mechanical forces, namely elevated ICP, in the development of papilledema [4] and associated retinal vascular changes resulting from compression of vessels by the swollen optic disk [5]. Hence, vascular morphological and structural parameters have the potential to be used as imaging biomarkers of IIH. Specifically of interest are vascular biomarkers signaling subtle changes in papilledema to improve clinical assessment and disease monitoring. Additionally, relating quantitative measures of vascular biomarkers to papilledema severity improves the understanding of the role of vasculopathy in IIH.

The elevated ICP results in increased cerebrospinal fluid (CSF) pressure in the optic nerve sheath, which leads to axoplasmic flow stasis and swelling of the optic nerve. This causes compression of the nearby retinal venules and local blood flow stasis, which ultimately exacerbates edema via leaking vasculature [6]. Studies on the vascular morphological and structural parameters in IIH have mainly focused on vessel diameter [7–10] and vessel density [11–15]. Specifically, a correlation between peripapillary capillary density and papilledema severity grading (Frisen score) has been reported [14]. Additionally, increased transmural pressure gradients across the vessel wall causes vessels and their downstream branches to become more tortuous according to the changes in blood flow [16]. Tortuosity alterations are dynamic, depending on the transmural pressure, and are considered reversible. Accordingly, retinal vessel tortuosity has also been shown to have potential as a biomarker of IIH [17] and is associated with optic nerve elevation [18]. However, to the best of our knowledge, the association between retinal vessel tortuosity and the severity of papilledema has not been reported. The purpose of the current study was to determine cross-sectional and longitudinal associations between papilledema severity and retinal vessel tortuosity in IIH.

## Methods

### Subjects

The retrospective study was approved by an Institutional Review Board of the University of Southern California in accordance with the ethical standards of the Declaration of Helsinki. Fifteen patients (24 eyes) with a clinical diagnosis of IIH and papilledema who met the modified Dandy criteria [19] were included in the study. Patients with ocular or neurological examination, MRI imaging, or CSF findings that were suggestive of etiologies other than elevated ICP (such as optic neuritis, anterior ischemic optic neuropathy, hypertensive optic disc edema, infectious diseases, or intracranial lesion) were excluded. Patients were treated based on their symptoms, retinal imaging findings, and visual function tests. All patients were counseled on weight loss. Twelve patients were treated with oral medication (acetazolamide and/or topiramate). Three of these twelve patients underwent bilateral optic nerve sheath fenestration. The remaining three patients did not receive medication. Based on clinical color retinal images centered on the optic nerve head, a neuro-ophthalmologist (author KKG) evaluated the severity of papilledema by assigning a Frisen score (FS):1 = C-shaped halo around the nerve head, 2 = circumferential halo around the nerve head, 3 = loss of major vessel(s) after leaving the disk, 4 = loss of major vessel(s) while still on the disk, and 5 = all vessels leaving the disk are compromised [20]. Eyes with FS ≤ 4 at first visit and lower FS at the second visit were included. Inter-visit differences in FS were calculated as: ΔFS = FS at visit 1 (FS1) – FS at visit 2.

### Image analysis

The vascular tortuosity measurement algorithms were developed in MATLAB (2015b, MathWorks, Inc., Natick, MA, USA) as previously described [21]. There are three steps to calculate retinal vessel tortuosity (RVT): vessel segmentation, centerline extraction, and first-order derivative algorithm.

### Vessel segmentation and centerline extraction

The methods for vessel segmentation and centerline extraction have been previously described [22]. Briefly, a Frangi filter [23] was applied for vessel segmentation, producing binary masks containing only the major retinal vessels, as previously demonstrated [24, 25]. The Frangi parameter settings, including scale range, threshold, and region size, can be manually adjusted in real-time based on the visualized segmentation results [21]. A trained operator performed segmentation to extract only the large vasculature around the optic nerve head. To extract vessel centerlines, a Euclidean distance transform was applied to the segmented vessels, generating a distance map in which local maxima indicate the central axis of each vessel. The algorithm automatically identifies vessel start- and end-points within a circumpapillary region of interest. Tortuosity was quantified from vessel centerlines. Arteries and veins were differentiated from color retinal images by a trained grader based on the relative size and difference in their light absorption properties.

### Vessel tortuosity measurement

The first-order derivative method (FD) for calculation of RVT was utilized, as previously described [21]. RVT was calculated according to the following equation:

$$\:RVT=\frac{\sum\:\left|y{\prime\:}\left(x\right)\right|}{{x}_{N}}$$

where, y’(x) is the first-order derivative computed pixel-wise from the image and $\:{x}_{N}$ is the chord length. RVT values were derived for each vessel and a mean value for arteries and veins was calculated for each eye. Inter-visit differences in RVT were calculated as: ΔRVT = RVT at visit 1 – RVT at visit 2.

### Vessel segment matching between visits

**Fig. 1:**
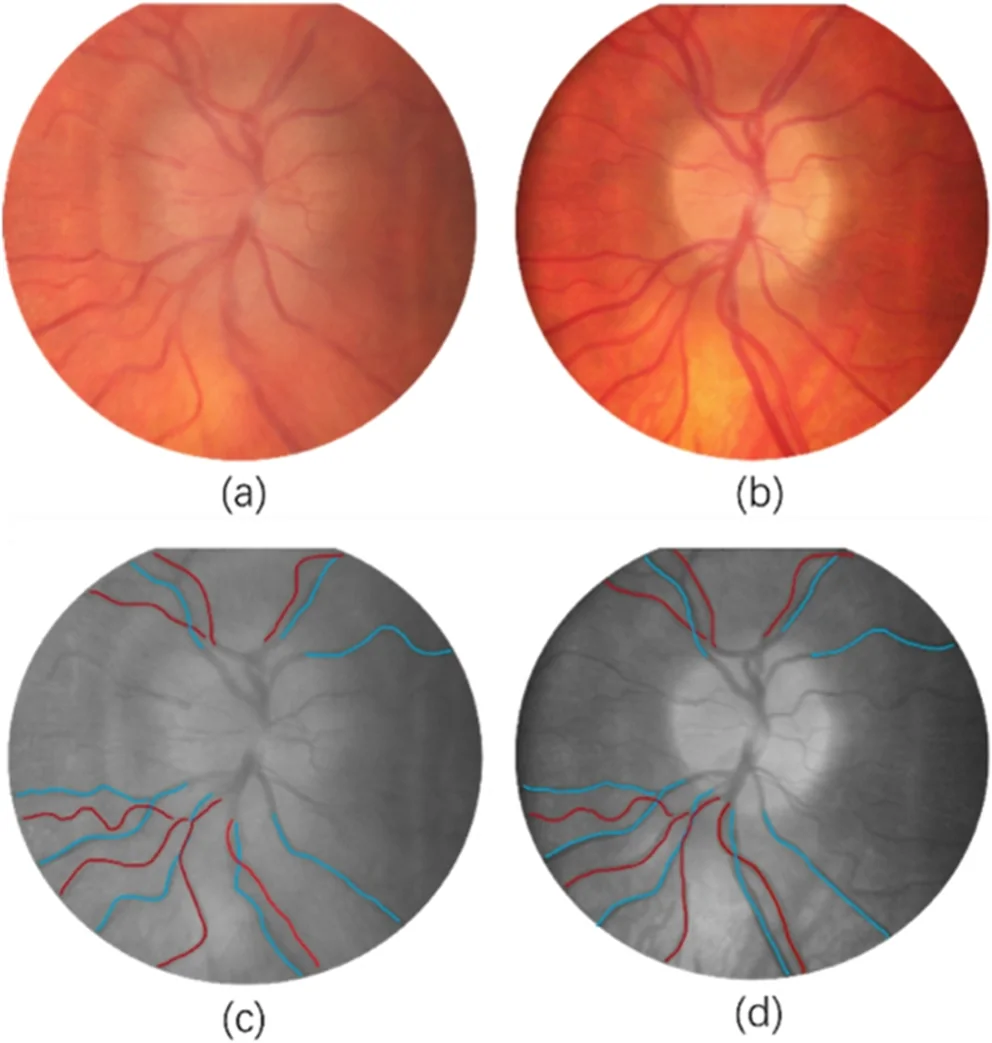
Retinal images from one subject: (**a**) first visit, Frisen score (FS) = 3; (**b**) second visit, FS = 0; **c**, **d**) Corresponding images displaying centerlines of segmented vessels for tortuosity measurements. Arteries and veins are shown in red and blue color, respectively

For the longitudinal assessment, vessel segments were manually matched for paired assessment of tortuosity changes. To spatially match the vessel segments at the two visits, the image with a smaller field of view was selected as the reference image. We then annotated the start- and end-points of the segmented vessels on the reference image, creating a vessel-guided mask. The start-point was defined as the intersection between the vessel centerline and the outer margin of the ONH, while the end-point was defined as the most distal location along the vessel centerline that remained clearly visible in the reference image. This mask was non-rigidly projected onto the image to be matched to locate the corresponding vessel end-points. In this way, anatomically consistent vessel segments were identified in both images, allowing for longitudinal vascular analysis. This approach emphasizes anatomical accuracy and repeatability, particularly in datasets with significant variation in field of view, image quality, or gaze angle between visits. An example of retinal images from one subject at two visits is shown in Fig. 1. In this example, the FS was reduced from 3 to 0 between visits, as visually depicted by a reduction in the severity of the papilledema.

### Statistical analysis

All statistical analyses were performed using IBM SPSS Statistics 29.0.1.0. No outliers were identified in the RVT values stratified by FS or vessel type, using the three-times interquartile range criterion. Generalized estimating equation models were generated to determine the associations of: (1) RVT with FS and vessel type; and (2) ΔRVT with ΔFS and vessel type, considering FS1 and inter-visit duration as covariates, and accounting for multiple measurements per subject. Parameter estimates (β) and p-values for the models were reported. Statistical significance was accepted at *p* ≤ 0.05.

## Results

Demographics and ocular characteristics of subjects are shown in Table 1. The inter-visit duration was 7.0 ± 7.8 months. Data from a total of 96 vessels (24 arteries and 24 veins at 2 visits) were available for analysis.

**Table 1:** Demographics of Study Participants

Variable	mean ± SD or Count	mean ± SD	
	Visit 1	Visit 2	*P*-value
Age (years)	32 ± 13		N/A
Sex (F/M)	14/1		N/A
Eye (OD/OS)	13/11		N/A
BMI	33.1 ± 8.4	33.7 ± 13.7	0.871
IOP (mmHg)	15.5 ± 3.7	15.4 ± 2.4	0.915
VA (logMAR)	0.14 ± 0.33	0.16 ± 0.35	0.410
RNFLT (um)	213 ± 102	135 ± 56	< 0.001

Mean ± standard deviation (SD) or count are presented. BMI: body mass index; IOP: intraocular pressure; VA: visual acuity; logMAR: log minimum angle of resolution; RNFLT: retinal nerve fiber layer thickness

### Association of Retinal Vessel Tortuosity and Frisen Score

Based on compiled data from both visits, there was no significant interaction between FS and vessel type on RVT (*p* = 0.668). There was no overall significant association between RVT and FS (*p* = 0.894), while RVT was significantly associated with vessel type (*p* = 0.018). Table 2 provides estimated means of RVT and parameter estimates for FS *≥* 1 with FS = 0 as reference and for veins compared to arteries. RVT was higher in veins than in arteries.

**Table 2:** Association of Retinal Vessel Tortuosity (RVT) and Frisen Score (FS)

FS	RVT				
	Estimated Mean	SEM	*N*	β	*P*-value
4	0.27	0.03	4	0.02	0.570
3	0.29	0.02	28	0.002	0.911
2	0.28	0.01	20	-0.007	0.513
1	0.27	0.02	26	-0.01	0.497
0	0.28	0.006	18	ref	
Vessel Type					
Veins	0.31	0.02	48	0.06	0.028
Arteries	0.24	0.01	48	ref	

RVT: retinal vessel tortuosity; FS: Frisen score; SEM: standard error of the mean; β: parameter estimate

### Association of Changes in Retinal Vessel Tortuosity (ΔRVT) and Frisen Score (ΔFS)

There was a significant interaction between ΔFS and vessel type on ΔRVT (*p* = 0.036). Table 3 lists estimated means of ΔRVT and parameter estimates for ΔFS *≥* 1 with ΔFS = 0 as reference and stratified by vessel type. Compared to ΔFS = 0, arterial ΔRVT was higher when ΔFS *≥* 1, while venous ΔRVT was higher when ΔFS = 3. Including the inter-visit duration as a covariate in the model did not change the statistical results.

**Table 3:** Association of Changes in Retinal Vessel Tortuosity (ΔRVT) and Frisen Score (ΔFS)

Vessel Type		ΔRVT				
	ΔFS	Estimated Mean	SEM	*N*	β	*P*-value
Arteries						
	3	0.05	0.02	4	0.06	0.003
	2	0.02	0.01	5	0.04	0.042
	1	0.03	0.008	5	0.05	< 0.001
	0	-0.02	0.008	10	ref	
Veins						
	3	0.07	0.01	4	0.05	0.005
	2	0.03	0.01	5	0.02	0.365
	1	0.02	0.01	5	0.00	0.808
	0	0.02	0.01	10	ref	

ΔRVT: change in retinal vessel tortuosity; ΔFS: change in Frisen score; SEM: standard error of the mean; β: parameter estimate

## Discussion

In the current study, based on compiled data from all levels of papilledema severity, RVT was shown to be higher in veins than arteries. This finding is supported by the knowledge that veins are more compliant than arteries, and thus more susceptible to pressure changes, and is consistent with previous studies [26, 27]. Based on compiled data from arteries and veins in the current study, papilledema severity was not shown to be associated with increased vessel tortuosity in a cross-sectional analysis. To the best of our knowledge, this is the first report of the association of papilledema severity with retinal vessel tortuosity. Several factors likely contributed to this finding. First, there are possible variations in resistance of the vessel walls to increased pressure and treatment response among subjects, such that the same papilledema severity may result in different levels of vessel tortuosity. Second, heterogeneity in disease duration and treatment protocol may have also increased the outcome variability. Third, data in a limited range of Frisen scores were included in the analysis, which coupled with subjective Frisen score grading, may have reduced the ability to find a significant association between papilledema severity and vessel tortuosity. Overall, future studies are needed to account for these variabilities and assess the relationship between papilledema severity and vessel tortuosity.

Longitudinal reductions in papilledema severity were shown to be associated with decreased retinal vessel tortuosity in both arteries and veins. Based on current literature, this is the first report of the association between improvement in papilledema severity score and retinal vessel tortuosity. The finding of decreased vessel tortuosity suggests mechanical stress on the vessels is diminished due to reduced papilledema. Compared to the cross-sectional analysis, the pairing of the vessel for the longitudinal analysis reduced inter-subject variability which provided a more robust assessment of the relationship between papilledema and vessel tortuosity.

Differences in association of changes in papilledema severity and vessel tortuosity according to vessel type were demonstrated. In retinal veins, the decrease in vessel tortuosity was only significant with large reductions in papilledema severity. Since retinal veins are considered to be more compliant and demonstrate higher pathological tortuosity than arteries, they may be more sensitive to changes in papilledema severity than arteries. However, during the recovery of papilledema, it is plausible that mechanical stress surrounding the retinal vasculature is relieved, veins—owing to their inherently high compliance but low elastic recoil—may exhibit delayed or incomplete restoration of their original geometry [28]. *In* vivo studies of the saphenous vein have demonstrated a clear hysteresis in the pressure–diameter curve [28]. Consistently, many experimental studies have observed incomplete venous recoil, which is attributed to the limited elastic content of venous walls [29–31]. Furthermore, under sustained mechanical high-pressure conditions, it is conceivable that blood vessels may undergo not only geometric changes in lumen diameter but also structural damage to the vessel wall. A reduction in venous elastic fibers may further diminish recoil capacity [32]. In contrast, arterial walls, which are richer in elastic fibers, may likely recover more effectively once the pressure is reduced [33]. Our findings indicate that any improvement in papilledema severity resulted in a reduction of retinal arteriolar tortuosity.

Clinically, RVT shows promise as a quantitative and objective vascular biomarker to complement existing papilledema assessments. RVT is a continuous variable and may be able to capture subtle vascular changes associated with a reduction in severity of papilledema, compared to the categorical and subjective grading of optic disc edema. Future studies are needed to establish the potential clinical application of RVT for quantitative monitoring of progression or treatment response of papilledema. Additionally, changes in RVT may reflect underlying alterations in ICP and ONH biomechanics, thereby providing insight into the IIH condition.

This study had limitations. First, small sample size, retrospective study design, variability in the disease duration, treatment regimen, and follow-up intervals introduced heterogeneity, which may limit the generalizability of the findings. Second, RVT was averaged over all arteries and veins in each eye which may have filtered out tortuosity variations among vessels. Third, tortuosity of only large peripapillary vessels was evaluated, limiting detection of changes in small vessels. Fourth, subjective assessment of papilledema severity based on Frisen score and potential for high inter-observer variability was also a limitation of the study. Finally, data on subretinal fluid accumulation and blood pressure were not available and may have affected reported RVT measurements. Future prospective studies in a larger cohort are needed to establish vessel tortuosity as a biomarker of papilledema.

Overall, an association between improved papilledema severity and decreased retinal vessel tortuosity in the peripapillary region was demonstrated. The findings show promise for vessel tortuosity as a biomarker of papilledema for monitoring disease progression and response to therapeutic interventions.

## Data Availability

The data that support the findings of this study are available from the authors, upon reasonable request.
